# Doenças Crônicas Pregressas e sua Relação com a Infecção por COVID-19

**DOI:** 10.36660/abc.20210859

**Published:** 2022-07-29

**Authors:** Bruna Redivo de Souza, Eliane Mazzuco, Layse Wiggers Kemper

**Affiliations:** 1 Universidade do Sul de Santa Catarina Tubarão SC Brasil Universidade do Sul de Santa Catarina – UNISUL, Tubarão, SC – Brasil

**Keywords:** Diabetes Mellitus/prevalência, Hipertensão/prevalência, COVID-19, Pandemia, Fatores de Risco, Estudos Epidemiológicos, Atenção Primária a Saúde, Sistema Único de Saúde (SUS)

**Prezado Editor,**

O assunto abordado no estudo é de grande relevância em se tratando do atual período pandêmico enfrentado mundialmente. Sabe-se que existe uma certa urgência na produção e divulgação de dados científicos e epidemiológicos sobre o novo Coronavírus. Sendo assim, pesquisas que ajudem a traçar o perfil da população mais vulnerável a essa doença trazem uma grande contribuição para que se evite um número ainda maior de óbitos e sequelas decorrentes da COVID-19.

Apesar do coronavírus infectar pessoas de todas as idades, existe uma prevalência de complicações entre dois grupos: os idosos e os que têm comorbidades preexistentes. Considerando este último grupo, a hipertensão arterial sistêmica (HAS) e o diabetes mellitus (DM) são dois dos principais fatores de risco para a mortalidade por COVID-19.^1^ Em concordância a este dado, um estudo sobre a multimorbidade dos brasileiros publicado em Cadernos de Saúde Pública (CSP), demonstrou que aproximadamente 72% dos pacientes internados em UTI por COVID-19 apresentavam doenças crônicas pregressas em comparação àqueles que não necessitaram destes cuidados intensivos (37%).^2^

Diante disso, evidencia-se um grande contingente de pessoas em risco da COVID-19 grave no país, reforçando que o perfil de comorbidades da população brasileira é um fator preocupante e que precisa ser levado em consideração. Nesse cenário, a adoção de intervenções não farmacológicas torna-se fundamental para a prevenção de casos graves da infecção,^2^ uma vez que muitos dos fatores agravantes são preveníveis, e garantir um estilo de vida mais saudável para a população refletiria de forma positiva no combate a pandemia.

Portanto, os estudos epidemiológicos são ferramentas importantes para caracterizar o comportamento típico da doença, assim como orientar as tomadas de decisões no âmbito das políticas públicas em saúde e vigilância epidemiológica.^3^ Assim, a estimativa apresentada é importante para planejar as estratégias de monitoramento das pessoas com morbidades crônicas e de prevenção no enfrentamento do novo coronavírus.^3^

Nesse contexto, o Sistema Único de Saúde (SUS) e a atenção primária à saúde, por intermédio da coordenação do cuidado pela Estratégia Saúde da Família, continuarão a ter papel relevante para amenizar as iniquidades sociais em saúde, por meio da prevenção da infecção pelo vírus e o manejo de condições crônicas e multimorbidade durante e após a pandemia.^2^
