# Supplementary material for: Doenças Crônicas Pregressas e sua Relação com a Infecção por COVID-19
Source: Arq Bras Cardiol. 2022 Jul 29;119(2):361–2. [Article in Portuguese] doi: 10.36660/abc.20210859 (PMC9363057; doi:10.36660/abc.20210859)
Supplement: Translation

# Translation: Previous Chronic Diseases and their Relationship with COVID-19 Infection

**Authors:** Bruna Redivo de Souza, Eliane Mazzuco, Layse Wiggers Kemper

**Affiliations:** 1 Tubarão SC Brazil Universidade do Sul de Santa Catarina – UNISUL, Tubarão, SC – Brazil

**Keywords:** Diabetes Mellitus/prevalence, Hypertension/prevalence, COVID-19, Pandemic, Risk Factors, Epidemiologic Studies, Primary Health Care, Unified Health System

**Dear editor,**

Due to the current global pandemic, the subject addressed is of great relevance. It is known that there is a certain urgency in the production and dissemination of scientific and epidemiological data about the new Coronavirus. Therefore, research that helps profile the population most vulnerable to this disease contributes to preventing an even greater number of deaths and sequelae resulting from COVID-19.

Although the Coronavirus infects people of all ages, complications are prevalent among two groups: the elderly and those with pre-existing comorbidities. Considering this last group, systemic arterial hypertension (SAH) and diabetes mellitus (DM) are two of the main risk factors for mortality from COVID-19.^1^ In agreement with this data, a study on the multimorbidity of Brazilians published in Cadernos de Saúde Pública (CSP), showed that approximately 72% of patients admitted to the ICU for COVID-19 had previous chronic diseases compared to those who did not need this intensive care (37%).^2^

Consequently, there is a large contingent of people at risk of severe COVID-19 in the country, reinforcing that the profile of comorbidities in the Brazilian population is a worrying factor that needs to be considered. In this case, the adoption of non-pharmacological interventions becomes fundamental for the prevention of severe cases of infection,^2^ since many of the aggravating factors are preventable, and ensuring a healthier lifestyle for the population would reflect positively on the battle against the pandemic.

Therefore, epidemiological studies are important tools for characterizing the typical behavior of the disease and guiding public policy decisions in health and epidemiological surveillance.^3^ Thus, the estimate presented is important to plan people’s monitoring strategies for chronic morbidities and prevention in the fight against the new Coronavirus.^3^

In this context, the Brazilian Unified Health System (SUS) and primary health care, through the coordination of care by the Family Health Strategy, will continue to have an important role in mitigating social inequities in health through the prevention of virus infection and management of chronic conditions and multimorbidity during and after the pandemic.^2^
