# Supplementary material for: Doenças Crônicas Pregressas e sua Relação com a Infecção por COVID-19
Source: Arq Bras Cardiol. 2022 Jul 29;119(2):361–2. [Article in Portuguese] doi: 10.36660/abc.20210859 (PMC9363057; doi:10.36660/abc.20210859)
Supplement: Related texts

# Reply: Carta-resposta

**Authors:** Carlos Souza, Lucas Santos, Jussara Baggio, Thiago Leal, Francisco Costa, Tânia Fernandes, Regicley Silva, Anderson Armstrong, Rodrigo Carmo

A transformação de dados em informações, de informações em conhecimento e de conhecimento em sabedoria, embora não seja uma tarefa fácil- *sobretudo em tempos de pandemia*-, é fundamental para a intervenção oportuna. Ainda mais quando essas intervenções podem salvar a vida de inúmeras pessoas *[o termo ‘pessoas’ aqui aludido tem o sentido geométrico do ser sujeito. São seres reais, concretos e donos de projetos de vida e de felicidade].* Produzir ciência é, portanto, um ato de compromisso com esses sujeitos. O bem tutelado é a própria vida – *nossa e dos nossos!*

No Brasil, desde o ano de 2006 vem sendo publicada a mais importante pesquisa sobre fatores de risco e proteção para doenças crônicas nas capitais dos 26 e estados e no Distrito Federal, totalizando 27 cidades (Vigitel- *Vigilância de fatores de risco e proteção para doenças crônicas por inquérito telefônico*).^1^ Os dados coletados em 2020, embora demonstrem avanços, ainda são preocupantes:

- a frequência de adultos (≥18 anos) fumantes nas 27 cidades foi de 9,5%;
- o excesso de peso foi (IMC ≥ 25 kg/m^2^) observado em 57,5% da população e a obesidade (IMC ≥ 30 kg/m^2^) em 21,5% dos indivíduos;
- a prática de atividade física no tempo livre equivalente a 150 minutos de atividade moderada por semana foi relatava por pouco mais de um terço da população (36,8%);
- a frequência de hipertensão arterial alcançou ¼ da população (25,2%); e
- a frequência de Diabetes *Mellitus* foi de 8,2%.^2^

O aumento da prevalência de fatores de risco e das próprias doenças crônicas é uma realidade preocupante, não somente no Brasil, mas em todo o mundo. Somente em 2019, 54,7% das mortes registradas no Brasil tiveram como causa as doenças crônicas não-transmissíveis.^3^ Esse cenário exige um esforço coletivo – *gestores, profissionais de saúde e sociedade civil* –, e intersetorial, envolvendo todos os níveis de atenção e de prevenção. Somente um amplo conjunto de políticas é capaz de impactar satisfatoriamente nesse cenário.

Considerando tal contexto, o Brasil lançou em 2021 o “*Plano de Ações Estratégicas para o Enfrentamento das Doenças Crônicas e Agravos não Transmissíveis no Brasil 2021-2030*”^4^com o objetivo de “*fortalecer a agenda de enfrentamento das DCNT, das violências e dos acidentes nas esferas federal, estaduais, municipais e do Distrito Federal, bem como pautar a promoção da saúde nas ações de saúde”.*^4^ Cabe salientar que o plano em tela está em consonância com as recomendações mundiais e adota um arrojado sistema de monitoramento dos indicadores.

O lançamento do plano dialoga com a necessidade de produção do conhecimento sobre a influência dos fatores de risco (obesidade, sedentarismo e tabagismo, por exemplo) e doenças crônicas no desfecho clínico de indivíduos com COVID-19, bem como o impacto da pandemia – *e suas medidas de controle* – na prevalência desses fatores de risco e doenças. Tem-se uma via de mão dupla, com muitos questionamentos a serem respondidos.

Por fim, ficamos honrados ao receber o comentário referente ao nosso texto^4^ e agradecemos pelo momento de discussão.

## References

[1] Mássimo EAL, Souza HNF, Freitas MIF. Doenças crônicas não transmissíveis, risco e promoção da saúde: construções sociais de participantes do Vigitel. Ciênc saúde coletiva.2015;20(3):679-88. DOI https://doi.org/10.1590/1413-81232015203.14742014.

[2] Brasil. Ministério da Saúde. Vigitel Brasil 2019: vigilância de fatores de risco e proteção para doenças crônicas por inquérito telefônico: estimativas sobre frequência e distribuição sociodemográfica de fatores de risco e proteção para doenças crônicas nas capitais dos 26 estados brasileiros e no Distrito Federal em 2019. Brasília – DF;2020 .137p.

[3] Brasil. Ministério da Saúde. Secretaria de Vigilância em Saúde. Departamento de Análise em Saúde e Vigilância de Doenças Não Transmissíveis. Plano de ações estratégicas para o enfrentamento das doenças crônicas e agravos não transmissíveis no Brasil. Brasília – DF; 2020. vol.1,122p.

[4] Santos LG, Baggio JAO, Leal TC, Costa FA, Fernandes TRMO, Silva RV, et al. Prevalência de Hipertensão Arterial Sistêmica e Diabetes Mellitus em Indivíduos com COVID-19: Um Estudo Retrospectivo de Óbitos em Pernambuco, Brasil. Arq Bras Cardiol.2021;117(2):416-22. DOI https: https://doi.org/10.36660/abc.20200885.10.36660/abc.20200885PMC839578934495244

# Reply

**Authors:** Carlos Souza, Lucas Santos, Jussara Baggio, Thiago Leal, Francisco Costa, Tânia Fernandes, Regicley Silva, Anderson Armstrong, Rodrigo Carmo

The transformation of data into information, from information into knowledge and from knowledge into wisdom, while not an easy task – especially in times of a pandemic – is fundamental for timely intervention. Even more so when these interventions can save the lives of countless people [the term ‘people’ alluded to here has the geometric meaning of being a subject. They are real, concrete beings and owners of life and happiness projects]. Producing science is, therefore, an act of commitment to these subjects. The protected good is life itself – our and ours!

In Brazil, since 2006, the most important research on risk and protection factors for chronic diseases has been published in the capitals of 26 states and the Federal District, totaling 27 cities (Vigitel- Surveillance of risk and protection factors for diseases chronicles by telephone survey).^1^ The data collected in 2020, although showing progress, is still worrying:

- the frequency of adult (≥18 years) smokers in the 27 cities was 9.5%;
- overweight (BMI ≥ 25 kg/m2) was observed in 57.5% of the population, and obesity (BMI ≥ 30 kg/m^2^) was observed in 21.5% of the individuals;
- the practice of physical activity in free time equivalent to 150 minutes of moderate activity per week was reported by just over a third of the population (36.8%);
- the frequency of arterial hypertension reached ¼ of the population (25.2%); and
- the frequency of Diabetes Mellitus was 8.2%.^2^

The increase in the prevalence of risk factors and chronic diseases themselves is a worrying reality, not only in Brazil but throughout the world. In 2019 alone, 54.7% of deaths recorded in Brazil were caused by chronic non-communicable diseases.^3^ This scenario requires a collective effort – managers, health professionals and civil society – and intersectoral, involving all levels of care and prevention. Only a broad set of policies can satisfactorily impact this scenario.

Considering this context, in 2021, Brazil launched the “Plan of Strategic Actions to Combat Chronic Diseases and Non-Communicable Diseases in Brazil 2021-2030”^4^ with the objective of “strengthening the agenda for combating NCDs, violence and accidents at the federal, state, municipal and Federal District levels, as well as guide health promotion in health actions”.^4^ It should be noted that the plan in question is in line with global recommendations and adopts a bold indicator monitoring system.

The launch of the plan dialogues with the need to produce knowledge about the influence of risk factors (obesity, sedentary lifestyle and smoking, for example) and chronic diseases on the clinical outcome of individuals with COVID-19, as well as the impact of the pandemic - and its control measures – on the prevalence of these risk factors and diseases. There is a two-way street with many questions to be answered.

Finally, we were honored to receive the comment regarding our text^4^ and thank you for the moment of discussion.
